# Using Values Affirmation to Reduce the Effects of Stereotype Threat on Hypertension Disparities: Protocol for the Multicenter Randomized Hypertension and Values (HYVALUE) Trial

**DOI:** 10.2196/12498

**Published:** 2019-03-25

**Authors:** Stacie L Daugherty, Suma Vupputuri, Rebecca Hanratty, John F Steiner, Julie A Maertens, Irene V Blair, L Miriam Dickinson, Laura Helmkamp, Edward P Havranek

**Affiliations:** 1 University of Colorado Denver School of Medicine, Department of Medicine, Division of Cardiology Adult and Child Consortium for Health Outcomes Research and Delivery Science Aurora, CO United States; 2 Kaiser Permanente Mid-Atlantic States Mid-Atlantic Permanente Research Institute Rockville, MD United States; 3 Denver Health and Hospital Authority Department of Medicine Denver, CO United States; 4 Kaiser Permanente Colorado Institute for Health Research Denver, CO United States; 5 University of Colorado Denver Adult and Child Consortium for Health Outcomes Research and Delivery Science Aurora, CO United States; 6 University of Colorado Boulder Department of Psychology and Neuroscience Boulder, CO United States; 7 University of Colorado School of Medicine Adult and Child Consortium for Health Outcomes Research and Delivery Science Denver Health and Hospital Authority, Department of Medicine Denver, CO United States

**Keywords:** hypertension, social values, African Americans, medication adherence, health care disparities

## Abstract

**Background:**

Medication nonadherence is a significant, modifiable contributor to uncontrolled hypertension. Stereotype threat may contribute to racial disparities in adherence by hindering a patient’s ability to actively engage during a clinical encounter, resulting in reduced activation to adhere to prescribed therapies.

**Objective:**

The Hypertension and Values (HYVALUE) trial aims to examine whether a values-affirmation intervention improves medication adherence (primary outcome) by targeting racial stereotype threat.

**Methods:**

The HYVALUE trial is a patient-level, blinded randomized controlled trial comparing a brief values-affirmation writing exercise with a control writing exercise among black and white patients with uncontrolled hypertension. We are recruiting patients from 3 large health systems in the United States. The primary outcome is patients’ adherence to antihypertensive medications, with secondary outcomes of systolic and diastolic blood pressure over time, time for which blood pressure is under control, and treatment intensification. We are comparing the effects of the intervention among blacks and whites, exploring possible moderators (ie, patients’ prior experiences of discrimination and clinician racial bias) and mediators (ie, patient activation) of intervention effects on outcomes.

**Results:**

This study was funded by the National Heart, Lung, and Blood Institute. Enrollment and follow-up are ongoing and data analysis is expected to begin in late 2020. Planned enrollment is 1130 patients. On the basis of evidence supporting the effectiveness of values affirmation in educational settings and our pilot work demonstrating improved patient-clinician communication, we hypothesize that values affirmation disrupts the negative effects of stereotype threat on the clinical interaction and can reduce racial disparities in medication adherence and subsequent health outcomes.

**Conclusions:**

The HYVALUE study moves beyond documentation of race-based health disparities toward testing an intervention. We focus on a medical condition—hypertension, which is arguably the greatest contributor to mortality disparities for black patients. If successful, this study will be the first to provide evidence for a low-resource intervention that has the potential to substantially reduce health care disparities across a wide range of health care conditions and populations.

**Trial Registration:**

ClinicalTrials.gov NCT03028597; https://clinicaltrials.gov/ct2/show/NCT03028597 (Archived by WebCite at http://www.webcitation.org/72vcZMzAB).

**International Registered Report Identifier (IRRID):**

DERR1-10.2196/12498

## Introduction

Black Americans have a higher prevalence of uncontrolled hypertension than white Americans, leading to disparities in cardiovascular outcomes [1-4]. Uncontrolled hypertension disproportionately affects black patients than white patients. A 10 mm Hg difference in systolic blood pressure (SBP) is associated with an 8% increase in stroke risk for white patients and a 24% increase in risk for black patients [5]. Targeting uncontrolled hypertension has the potential to improve health outcomes for black patients.

The Institute of Medicine, World Health Organization, and others have identified poor adherence to medications as the most significant, modifiable contributor to uncontrolled hypertension [6-11]. The prevalence of poor adherence to antihypertensive medications ranges from 43% to 78%, with approximately 50% of hypertensive patients discontinuing the use of their medications after 1 year [11,12]. Poor adherence to antihypertensive medications is associated with poor outcomes and improving adherence reduces blood pressure (BP) [9-11,13-16]. Adherence rates are lower in black than white patients, with hypertension, and nonadherence has been shown to contribute to racial differences in hypertension control [7,17-20]. Targeted interventions to improve adherence in black patients have the potential to reduce racial disparities in hypertension outcomes.

**Figure 1:**
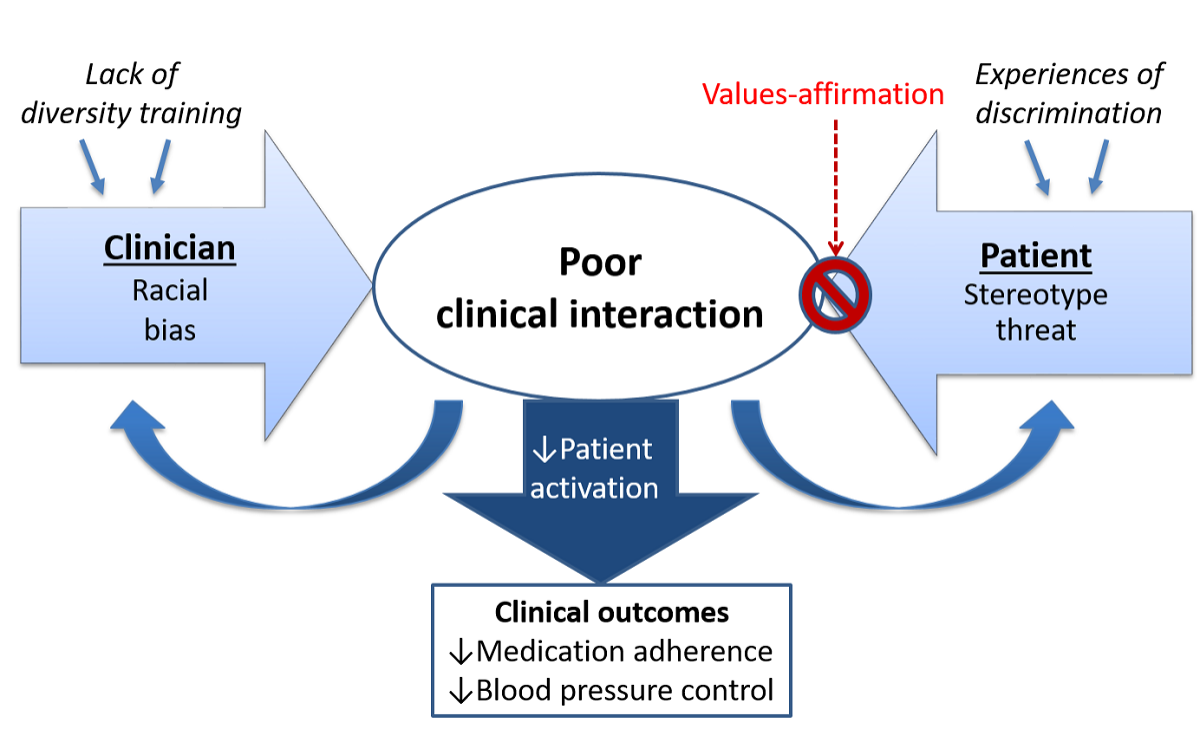
Conceptual model for reducing adherence disparities via targeting stereotype threat.

Stereotype threat may contribute to the racial disparity in adherence. Stereotype threat occurs when cues in the environment (such as visiting a white doctor’s office) trigger the threat of confirming, as self-characteristic, a negative stereotype about one’s group [21]. Although any individual may experience stereotype threat, black patients are at a greater risk due to widespread negative stereotypes and past experiences of discrimination [22-37]. In the clinical setting, black patients report stereotype threat related to being viewed as unintelligent, *second class citizens*, and unworthy of good care [21,36,38-40]. Stereotype threat triggers psychological and physiological responses including reduced memory capacity, impaired communication, disengagement, and reduced motivation [41,42]. Stereotype threat may contribute to racial disparities in adherence to treatment by hindering a patient’s ability to process information and actively engage in a discussion about their health during a clinical encounter [21,43]. On the basis of this poor clinical interaction, the patient may feel less activated to adhere to treatment recommendations [21,44]. Therefore, interventions targeting stereotype threat have the potential to reduce disparities in adherence and potentially in BP control among populations that experience widespread discrimination (Figure 1).

Interventions based on values affirmation have been shown to reduce stereotype threat and decrease racial disparities in academic outcomes [45]. These interventions typically ask participants to write about their core values, such as family, religion, or art [45]. By focusing on values that are important to them, values affirmation helps people to view themselves as worthy, effective, and able to control important outcomes despite perceived threats to oneself (eg, stereotype threat) [46-48]. Values-affirmation interventions have been associated with reduced stress and improved academic performance among stigmatized group members [49-53]. Cohen et al demonstrated that participation in an affirmation exercise at the beginning of the academic term reduced racial achievement gaps among black children by 40%; these effects were sustained 2 years later [49,50]. Our pilot work among 99 black patients at a single clinic analyzed the audiotaped patient-provider interactions and demonstrated that values affirmation significantly improved patient and clinician communication [54]. However, whether the intervention improves clinical outcomes such as adherence has not been evaluated. Furthermore, as the intervention was only conducted among black patients, our pilot study cannot determine whether values affirmation is targeting stereotype threat related to race or the general threat of illness. On the basis of the evidence supporting the effectiveness of values affirmation in educational settings and our pilot work demonstrating improved patient-clinician communication, we hypothesize that values affirmation disrupts the negative effects of stereotype threat on the clinical interaction and can reduce racial disparities in medication adherence and subsequent health outcomes.

We are conducting the Hypertension and Values (HYVALUE) study—a randomized controlled trial (RCT) in black and white patients with uncontrolled hypertension—to compare a values-affirmation intervention with a control exercise in 3 health care systems. Our primary objective is to examine whether patients in the intervention condition experience improvements in medication adherence relative to the control condition and compare these effects by race. Our secondary objective is to compare the intervention effects on BP over time, time for which BP is under control, and treatment intensification. Finally, we seek to determine moderators and mediators of intervention effects on patient outcomes over time.

## Methods

### Study Design and Setting

The HYVALUE study is designed as a patient-level, randomized, controlled, double-blinded, multicenter trial in primary care clinics in the United States. Clinics reside within 3 large health care systems that care for diverse populations: Denver Health Medical Center, Kaiser Permanente Colorado, and Kaiser Permanente Mid-Atlantic States. The study was registered on ClinicalTrials.gov on January 23, 2017 (NCT03028597). Recruitment began in February 2017 and is anticipated to continue through 2020 at medical centers in Colorado and Maryland. Enrollment is currently occurring in 10 clinics and we will add more clinics as needed to meet recruitment targets. As of December 10, 2018, a total of 623 patients are enrolled and a total of 1130 are planned.

### Study Population and Recruitment

The HYVALUE study is enrolling self-identified black and white patients who have uncontrolled hypertension, who meet all eligibility criteria, and who consent for the study. Patients with a diagnosis of hypertension with an upcoming clinic visit in a participating primary care clinic are screened on a regular basis for inclusion criteria using the electronic health record (EHR). Diagnosed hypertension is defined as having an outpatient visit in the past 24 months with a primary or secondary ICD-10 (International Classification of Diseases, 10^th^ revision) code diagnosis of hypertension. Uncontrolled BP is defined as having SBP ≥140 mm Hg or diastolic blood pressure (DBP) ≥90 mm Hg at least once during the preceding 12 months [54]. We have chosen a broad definition of uncontrolled BP as over 25% of patients with previously controlled BP lack BP control over the following year, likely due to nonadherence [55,56].

Additional inclusion criteria are age 21 years or above, self-described race and ethnicity as non-Hispanic black or non-Hispanic white, currently taking antihypertensive medications that are filled within patients’ health system’s pharmacy, and the ability to read and write English. Patients who are currently pregnant, have pregnancy-related hypertension, or have end-stage renal disease are excluded as not being representative of the broader population of patients with chronic hypertension. Patients are also excluded if they are unable to provide consent.

Patients meeting inclusion criteria are identified by data analysts from each site’s EHR and eligible patient lists are provided to the site research assistants on a weekly basis. Research assistants invite eligible patients to participate via telephone. The study team has implemented contingency strategies for meeting our target sample size. Such strategies have included adding recruitment locations in each health system, reallocating site-level resources toward recruitment efforts, and sending invitation postcards and emails to eligible patients before phone contact to introduce and increase interest in the study. Telephone contact is made before patients’ scheduled appointments to describe the study and answer any questions. If patients express interest in the study, eligibility is confirmed and patients are asked to arrive 1 hour before their clinician’s appointment to complete the baseline enrollment (index) visit (described below). Patients are asked to bring all BP medication bottles with them to appointments and are compensated for their time with a $20 (US) gift card at each visit.

### Study Protocol and Randomization

At the index visit, the on-site research assistant obtains informed consent and measures BP in accordance with policies set at each participating clinic. BP measurements are taken by standard clinic-grade calibrated automatic monitors at each site. To standardize methods across sites, BP is measured using the right arm, while patients are relaxed with both feet on the floor, using a well-fitting and calibrated cuff per guideline-recommended procedures [57]. A second BP measurement is taken at least 30 seconds after the first. Data are entered into the study database in real time and research assistants are prompted to take a third measurement when there is a large discrepancy between the first 2 measurements (at least 20 mm Hg systolic or 10 mm Hg diastolic). As part of the index visit, research assistants count the pills in each patient’s BP medication bottles and the patient completes surveys including (1) a demographics questionnaire, (2) a measure of past experiences of discrimination [58-62], and (3) a self-reported adherence measure [63].

**Figure 2:**
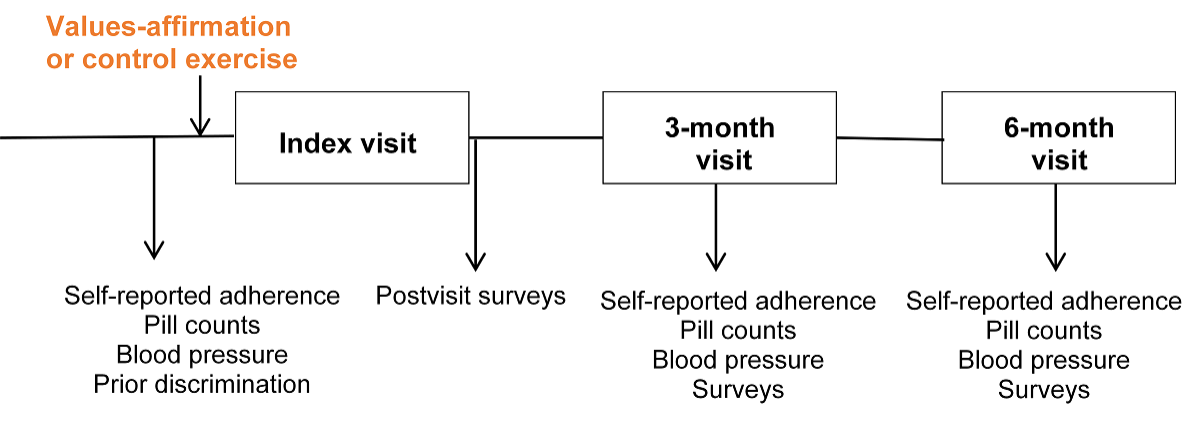
Study protocol flowchart.

After patients complete the previsit surveys, they are given a prerandomized, consecutively numbered packet containing either an intervention or control version of the values-affirmation exercise. Packets are randomized into separate series by race and medical center, using block randomization with random block size to ensure balanced randomization. The randomization was created by the data coordinating center analyst before the initiation of patient recruitment using SAS 9.4 (SAS Institute, Cary NC). The randomization lists for each site are stored in an access-restricted electronic folder and neither the research staff nor clinic staff know the patient’s study condition. Study packets are prepared by staff who are not involved in recruitment or enrollment. Each study packet contains a second, unlabeled envelope inside with either the intervention or control writing exercise. On-site research assistants provide general instructions that apply to both the intervention and control exercises. The patients complete the writing exercise on their own and are reminded not to unblind study staff to their condition. After completion of initial study activities, patients then proceed to their scheduled appointment with their clinician (Figure 2).

Following the appointment with their clinician, patients complete postvisit surveys including (1) a measure of patient activation [64]; (2) a measure of attitude, perceived social norms, perceived behavioral control, and emotion toward BP control [65,66]; and (3) a measure of satisfaction with their clinician [67]. Patients are contacted to schedule 3- and 6-month follow-up visits and, where possible, visits are scheduled during preexisting follow-up appointments scheduled with clinicians. Research assistants call patients 1 or 2 days ahead of time to remind them of the upcoming appointments. At 3 months, patients complete a follow-up study visit that includes measures of BP, pill counts, self-reported adherence, patient activation, and attitudes. Patients return at 6 months for a visit identical to the 3-month study visit (Figure 2). When patients choose to discontinue further study visits, are unreachable, fail to attend follow-up visits, or otherwise deviate from the study protocol, we continue to collect adherence and BP data over time via electronic pharmacy fill and the EHR.

### Values-Affirmation Intervention

**Figure 3:**
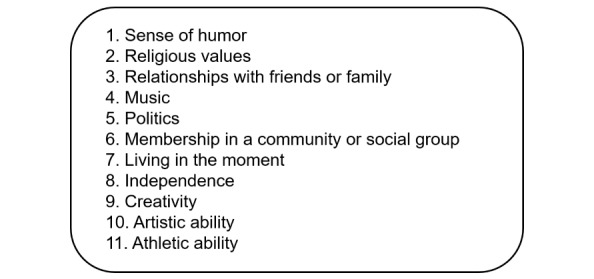
List of values for the intervention and control exercises.

All patients are randomized to either the values-affirmation intervention or control writing exercise. When the research assistants introduce the intervention tasks to the patient, they remain available for questions but otherwise do not participate in the completion of the task and remain blinded to the instructions and content of the exercise. The intervention task asks patients to reflect on a list of 11 personal values or self-defining skills and circle the 2 or 3 that are *most important to them* or that characterize them best (Figure 3). Next, patients are asked to think about the times when the values chosen were important and then write a few sentences to describe why they were important. Finally, the self-affirmation of values is reinforced by asking patients to indicate their level of agreement with 4 statements concerning the selected values: (1) *these values have influenced my life*, (2) *in general, I try to live up to these values*, (3) *these values are an important part of who I am*, and (4) *I care about these values*.

The control writing exercise asks patients to circle the 2 or 3 values that are *least important to them*. Control patients receive the same instructions to write a few sentences about the values chosen but are asked to describe when and why these least important values might be *important to* *someone else*. The final rating task asks patients to indicate their level of agreement with slightly altered statements: (1) *these values have influenced some people*, (2) *some people may try to live up to these values*, (3) *these values may be important to some people*, and (4) *some people care about these values*. After the task, patients place their responses in an envelope, seal them, and return them to the research assistant.

### Outcomes

The primary outcome is medication adherence over time. To address limitations inherent to each measure of medication adherence, 3 measures that differ in the behavior measured and time frame of observation are collected. The first measure of adherence reflects the intent to take a medication based on electronic pharmacy fill data. Pharmacy fill–based adherence assesses the proportion of days over the period of observation for which a patient obtains antihypertensive medications [68-73]. Pharmacy fill–based adherence will be calculated for each antihypertensive drug in the regimen and averaged across drugs into a summary measure of adherence for the entire drug regimen using the method developed by Steiner et al [68]. We will calculate pharmacy fill–based adherence for the 12 months before the index visit, 0 to 3 months, and 3 to 6 months after the index visit. The second measure of adherence is based on self-report of pill taking behavior over the previous 7 days [74]. This validated self-report instrument [63,75] is administered at each study visit. The third measure of adherence is based on pill counts that estimate pill taking behavior since the last medication refill. At each study visit, the research assistant enters the antihypertensive medication name, prescription fill date, number of pills prescribed, number of pills the patient should take every day, and recorded number of pills in the bottle into the research database. An embedded algorithm calculates pill count–based adherence for each medication [73]. All measures of adherence will be analyzed separately as primary outcomes. Medication adherence over time will be compared for intervention versus control patients and by race, as described below. Secondary outcome analyses will consider a composite adherence variable that takes missing data into account.

Secondary outcomes include SBP and DBP over time, time for which BP is under control (the proportion of time over follow-up with a BP ≤140/90 mm Hg) [57], and treatment intensification, which will be calculated by subtracting the number of expected intensifications (number of visits after enrollment with a BP ≥140/90 mm Hg) from the number of observed intensifications (either an increase in dose or addition of a new medication class) and then dividing this difference by the number of office visits over the study period [76].

### Patient Moderators and Mediators

We will assess patients’ prior experiences of discrimination as a potential moderator of the intervention effects [58-62]. We are using a 4-item questionnaire about prior experience of discrimination modified from prior scales measuring lifetime discrimination related to health care [58-62]. Responses are on a 5-point Likert scale ranging from *never* to *all of the time*.

Cognitive factors will be explored as mediators [77] of the intervention effect on adherence, including patient activation [64,78,79], patient impressions of their visits [67], and constructs from a modification for hypertension of the theory of planned behavior (attitude, perceived social norms, perceived behavioral control, and anticipated emotion toward managing their BP) [65,66].

Patient activation is measured by a low-literacy version of the 13-item instrument developed by Hibbard, the Patient Activation Measure (PAM) [64]. Increases in the PAM are associated with increased self-management behavior including adherence [78-80]. Patient activation measured with the PAM is lower for black patients than it is for white patients and is therefore hypothesized to be a contributing factor to racial health disparities [81].

Patients’ impressions of their visits are measured using Barr’s 4-item clinician-specific modification of the Medical Outcomes Study Visit Satisfaction Questionnaire, which is sensitive to differences in patient satisfaction by race [67].

Patient attitudes regarding their BP are measured by relying on constructs from the Theory of Planned Behavior, which posits behavior intention as the most proximal predictor of behavior and that intention is influenced by an individual’s attitude toward the behavior, the perceived social norms surrounding the behavior, and an individual’s perceived control over the behavior [82]. Taylor et al modified this theory by adding the concept of desire for the behavior and its consequences, which in turn is influenced by the anticipated positive and negative emotions associated with the behavior [65]. Perugini and Bagozzi tested the theoretical constructs in patients with hypertension [65,66]. We have adopted the questions from this study to assess patient attitudes, norms, perceived control, and emotions around BP management. We have added an additional question to include physicians’ opinions in the patients’ perception of social norms: *I care what my doctor thinks about my efforts to control my BP*.

### Clinician Substudy

As the clinical encounter may also be affected by clinician factors, we are conducting a substudy with clinicians. The primary aim of this substudy is to evaluate clinician factors that may moderate the relationship of the intervention with patient adherence including clinician implicit racial bias, prior cultural awareness training, and self-efficacy regarding caring for black patients*.* These measures were chosen based on literature suggesting that these factors influence the quality of the patient-clinician interaction and are associated with differences in racial attitudes among health care clinicians [83-85].

Clinician measures include the Black-White Implicit Association Test (IAT), which uses reaction times to assess the strength of automatic associations between race (black and white) and evaluations (eg, positive words and negative words). Results indicate that participants have an implicit preference for white over black individuals if they are faster to categorize words when white faces and positive words share a response key and black faces and negative words share a response key, relative to the inverse. The larger this performance difference, the stronger the implicit bias. The IAT has been widely validated, is reliable over time, and is associated with discriminatory judgments and behaviors [86-93]. The IAT has been used in the health care setting to measure clinician bias [20,94]. We also ask participating clinicians about past exposure to cultural awareness or diversity training and the degree to which they feel (1) *prepared to care for a patient who identifies as African American*, (2) *skilled about overcoming unintended or implicit racial bias related to African American patients*, and (3) *skilled about developing a positive relationship with African American patients* [83].

All clinicians at the participating clinics are invited to complete the clinician surveys; patient participation is independent of whether their clinician participates. Clinician recruitment began in November 2017.

### Sample Size Justification

For our primary analysis, we assume a 3-level model, with patients nested within clinicians and clinicians nested within clinics. Assuming comparable variation in adherence and alpha=.05, a sample size of 960 participants and 1:1 enrollment by race (240 in each study condition, for each racial group; approximately 480 black and 480 white patients) will be required to detect a 0.26 effect size difference in adherence between any 2 cells with a power of 80%. A 0.26 effect size is approximately a 4.7% absolute difference in adherence scores (assuming SD=18.1) between black and white patients using the pharmacy fill data. A 2:1 enrollment ratio for black versus white patients (approximately 640 black and 320 white patients) would provide >80% power to detect a 0.28 effect size difference or an absolute difference in adherence scores of 5.1% between black and white patients receiving the intervention. We anticipate enrolling an additional 170 patients to compensate for attrition, for a projected final sample size of 1130. As randomization is at the patient level, the intraclass correlation coefficient for patients nested within clinicians will have a negligible effect on power.

For the secondary outcome measure of BP, this sample size has a power of 80% to detect a 4.7 mm Hg difference in SBP between any 2 cells (based on data from patients meeting criteria for another study). For perspective, in a study of a pharmacist-led multimodal intervention, an increase in adherence from 62% (n=179) to 97% (n=159) at 6 months (a 35% absolute increase) was associated with a decline in SBP from 133.2 mm Hg to 129.9 mm Hg (a 3.3 mm Hg absolute decrease) [16]. Thus, the study is likely underpowered to detect a difference in SBP through adherence change alone. Given the established link between higher adherence rate and improved clinical outcomes, we believe a study powered to detect an adherence difference will be sufficient evidence to move this intervention forward into clinical practice [71,95,96]. Sample size calculations were generated using SAS 9.4 (SAS Institute, Cary NC).

### Statistical Analysis

Data analysis of the trial results will begin when all enrolled patients have completed follow-up. Descriptive statistics (chi-square and *t* tests) will be computed to determine whether there are differences between eligible patients who have enrolled and those who have not enrolled, between patients randomized to different study arms, or between dropouts and nondropouts.

Before beginning analyses of outcomes, we will examine the data to determine whether patterns of missingness are ignorable (Missing Completely at Random or Missing at Random) or nonignorable (Missing Not at Random) [97-99]. We will employ likelihood–based methods that utilize all available data, adjusting for covariates that are associated with missingness. If missingness is nonignorable we will employ pattern mixture models [100]. Sensitivity analyses will be carried out using multiple imputation approaches.

The 3 primary measures of adherence will be evaluated in separate analysis. The pharmacy fill– based adherence measure is continuous; in the event that normality assumptions are not met, we will use transformations to normalize distributions. For the primary comparisons, we will employ intent-to-treat analyses, although we expect few or none of the randomized patients to not complete an exercise. We will create models with adherence over time as the dependent variable and assignment to values affirmation or control exercise as the treatment variable. We will examine the effect of values affirmation on change in overall medication adherence and by racial groups. We will use general linear mixed models with random effects for patient and clinic to determine whether change in adherence differs by study arm and race. Fixed effects will include time (preintervention, 0 to 3 months, and 3 to 6 months), race, study arm, all 2-way interactions, and the 3-way interaction. The 3-way interaction term (time×race×study arm) will test for a differential intervention effect by race. Models will then be assessed after including potential moderator variables (eg, patients’ prior experiences of discrimination) and similarly for potential mediator variables (eg, patient activation, attitude, social norms, perceived behavioral control, and anticipated emotion) [101]. Analysis using the pill count measure of adherence will be similar to the methods described for the pharmacy fill measure. For analysis using the self-reported adherence measures, we will dichotomize the groups into nonadherent (score ≥2 on any *extent* item) and adherent. For this analysis we will use a mixed effects logistic regression model (generalized linear mixed model).

For the secondary outcome of BP, we will examine differences in SBP over time by intervention group and race using longitudinal mixed effects models with random intercepts and slopes. Similar to the analysis described above, the 3-way interaction term (time×study arm×race) will test for differential intervention effectiveness (differences in slopes).

Given evidence suggesting bias effects vary by age, the intervention effect will be compared by patient age [84]. Other a priori planned subgroup comparisons for hypothesis generation include patient gender, number or years living in the United States, socioeconomic status, BP control status, clinician race, and number of medications.

In the clinician substudy, among the patients whose clinicians submitted a survey, we will explore whether the collected clinician variables (implicit racial bias, past cultural competency training, and self-efficacy) moderate the relationships. We will also include patient gender and clinician race as possible moderating variables.

All hypothesis tests will be 2-sided with alpha=.05. Statistical analysis will use SAS 9.4 (SAS Institute, Cary NC).

### Study Ethics

The principal investigators are ensuring the conduct of and oversight for the study according to National Institutes of Health (NIH) and national policies. The institutional review boards (IRBs) for the University of Colorado School of Medicine and Denver Health (the Colorado Multiple Institutional Review Board), Kaiser Permanente Colorado, and Kaiser Permanente Mid-Atlantic States have reviewed and approved the study. Continuing review of study enrollment and procedure is required annually by each IRB and adverse events are required to be reported on an ongoing basis.

Patient informed consent is obtained in a clinic examination room or other private area to allow the process to be private and confidential. Following elucidation of the nature, risks, and possible benefits of the study, patient participants are asked to sign written informed consent as approved by the IRB for their respective health system. The consent process explicitly states the decision on whether or not to participate will in no way affect current or future care. Furthermore, the consent process is carried out by research staff, not clinic personnel, further decoupling the research and patients’ usual care.

For the clinician substudy, clinician informed consent is administered at the survey website. After using their unique study identifier to log on to the study website, clinicians are informed about the goals and procedures of the research including information about both their direct participation (ie, completing the online survey) and the participation of their patients (ie, associating their attitudes data to patients’ data). No signature or any other paperwork is collected from the clinician participants, thereby eliminating identification from sources outside of study personnel.

### Data Management and Monitoring

The risk of inadvertent or unauthorized release of confidential participant information is prevented in a number of ways. As a general step, all paper documents are stored in a locked file whose sole purpose is storage of clinical research material. Patient survey data are collected and managed using REDCap hosted at the University of Colorado [102]. REDCap is a secure, Web-based application designed to support data capture for research studies. Each health system maintains extensive patient clinical data in a series of standardized virtual data warehouses that are available for research applications; deidentified data from each site will be transferred to the primary analytic site via a secure encrypted website at the time of analysis. Clinician survey data are collected using a Web server at the University of Colorado, which uses encryption for data transfer between the respondent’s computer and the research server. All data will be deleted from the site servers 5 years following completion of analyses.

In addition to multisite IRB oversight, a data safety monitoring board (DSMB) is in place. The board has 3 members with expertise in biostatistics, primary care, and cardiology who are outside of the investigative team. The DSMB reviewed the protocol before the implementation and will continue to meet at least every 12 months throughout the study. The board reviews evidence of any study-related adverse events, data quality and completeness, and adherence to the protocol. DSMB meeting summaries are reported to each site IRB and to the project funder. Interim analyses and protocol revisions are currently not planned but will be conducted upon the request of the board.

Study results will be disseminated to researchers and the public via publication and conference presentations, to participating clinicians via site-level meetings, and to other stakeholders as appropriate.

## Results

This RCT was funded by the National Heart, Lung, and Blood Institute, and the results can provide evidence for a low-resource intervention that may reduce health care disparities across health care conditions and populations. Planned enrollment is 1130 patients. Data collection is ongoing and the results are expected in early 2021. On the basis of the evidence supporting the effectiveness of values affirmation in educational settings and our pilot work demonstrating improved patient-clinician communication, we hypothesize that values affirmation disrupts the negative effects of stereotype threat on the clinical interaction and can reduce racial disparities in medication adherence and subsequent health outcomes. If successful, a values affirmation intervention could reduce disparities in hypertension outcomes.

## Discussion

The primary objective of the HYVALUE study is to assess the effect of a values-affirmation exercise on medication adherence of black and white patients with hypertension, using a blinded RCT. Widespread exposure to discrimination among minority populations increases stereotype threat, making intervention even more important. Stereotype threat may impair communication between minority patients and their clinicians because of increased stress, resulting in interactions that are less successful at enhancing patient engagement with hypertension treatment, which in turn could result in lower rates of adherence with antihypertensive medication (Figure 1). This notion implicates stereotype threat as a cause of poor health outcomes for minority patients.

We hypothesize that a values-affirmation exercise performed before a patient-clinician visit reduces the impact of stereotype threat, ultimately resulting in better adherence to prescribed antihypertensive medications. The HYVALUE trial includes a sample of patients with uncontrolled hypertension from 3 health care systems, who are randomized to perform a brief values-affirmation writing exercise or a control writing exercise before a scheduled clinic visit. The primary outcome is adherence to antihypertensive medications and the secondary outcomes are BP over time, time for which BP is under control, and treatment intensification. To better understand the effects of the intervention, we are measuring theory-driven potential mediators and moderators. If the intervention improves adherence, this study will be the first to implicate stereotype threat related to race directly in the genesis of health disparities and will provide evidence for a low-resource intervention that could substantially reduce health disparities across a wide range of conditions and populations.

The HYVALUE study is innovative for the following reasons. First, we use a unique intervention that has been widely successful at reducing racial disparities in other domains [48-52]. Values affirmation has been shown to improve communication between black patients and white clinicians; however, no study has examined whether the intervention improves clinical outcomes [103]. Second, the theoretical model (Figure 1) on which this proposal is based is supported by patient engagement theory [104]. The HYVALUE study will be the first study to evaluate whether values affirmation improves patient activation and reduces racial disparities in clinical outcomes. Third, compared with existing interventions to improve adherence, values affirmation is significantly less time and resource intensive, enhancing the potential for the intervention to be embedded in primary care [105]. Finally, stereotype threat is not specific to hypertension outcomes or black patients. Health care disparities have been demonstrated across numerous disease states and minority groups [106]. Therefore, a simple intervention targeting a common mechanism has the potential to significantly reduce a wide range of health disparities.

### Conclusions

The HYVALUE study moves beyond documentation of race-based health disparities towards testing an intervention. We focus on a medical condition – hypertension – that is arguably the single greatest contributor to mortality disparities for blacks. Our pilot data suggest a values-affirmation intervention improves patient-clinician communication. The HYVALUE trial is comparing a brief values-affirmation writing exercise with a control writing exercise among black and white patients with uncontrolled hypertension. If successful, this trial will provide evidence for a low-resource intervention that has the potential to substantially reduce health care disparities across a wide range of health care conditions and populations.

## Abbreviations

BP: blood pressure
DBP: diastolic blood pressure
DSMB: data safety monitoring board
EHR: electronic health record
HYVALUE: hypertension and values
IAT: implicit association test
IRB: institutional review board
NIH: National Institutes of Health
PAM: patient activation measure
RCT: randomized controlled trial
SBP: systolic blood pressure
